# Unlocking the Potential of Gold as Nanomedicine in Cancer Immunotherapy

**DOI:** 10.3390/jnt5020003

**Published:** 2024-04-30

**Authors:** Panangattukara Prabhakaran Praveen Kumar, Maggie Lee, Taeho Kim

**Affiliations:** Department of Biomedical Engineering, Institute for Quantitative Health Science and Engineering, Michigan State University, East Lansing, MI 48824, USA

**Keywords:** nanotechnology, nanomedicine, gold nanoparticles, cancer immunotherapy, photothermal therapy, photodynamic therapy, radiation therapy, sonodynamic therapy

## Abstract

Nanotechnology advancements have resulted in many sensors and devices for biomedical applications. Among the various nanomaterials, gold nanoparticles (AuNPs), due to their size, shape, biocompatibility, and unique plasmonic property, are an excellent candidate for many biomedical applications. AuNPs, known for their easy surface modifications, robust nature, and photothermal activities, find application in drug delivery and cancer treatment studies. In this review, we are highlighting the recent trends in using AuNPs as nanomedicine for cancer immunotherapy. Cancer immunotherapy not only eliminates the primary tumors but also allows for the treatment of metastasis along with the recurrence of the tumor. AuNPs possess tissue-specific delivery functions that depend on the tunability in size and surface functionalization of AuNPs. AuNPs can be used to activate the tumor’s immune defense ability, or they can be used to enhance the anti-tumor immune response. Understanding the interaction of the tumor environment and nanobiomedicine is very important. In the present review, we give an idea of the mode of action of AuNPs and various combinations of therapies for cancer immunotherapy.

## 1. Introduction

Cancer has become one of the major global health problems in recent years [1,2]. Enormous effort has been devoted by researchers and medical teams to treat various forms of cancer. Conventional treatment methods like surgery, chemotherapy, and radiotherapy possess side effects, and the recurrence of cancer is a threat to fully utilizing such methods [3]. Moreover, the effect of treatments varies depending on the type of cancer, and the side effects caused by therapies must be taken care of for clinical point-of-care applications. Moreover, the existing methods are expensive, and, according to the EU and the US, the estimated expenditure for cancer treatment is $173 billion as of 2020 [4,5]. Therefore, developing new techniques and treatment methods that are economical and promising for the diagnosis and treatment of cancer is required.

**Figure 1.**
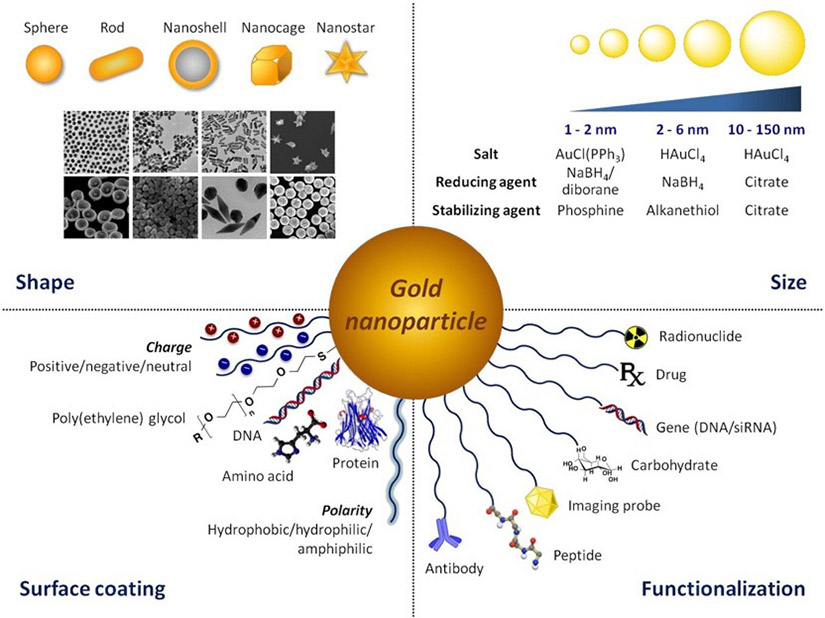
Represents the synthetic versatility and functionalization properties of AuNPs. The size and shape of AuNPs can be fine-tuned using different synthetic procedures, and reducing or stabilizing agents. Reproduced with permission from [36]. Copyright 2017, Elsevier.

Over the last few years, advancements in nanoscience and nanotechnology have overcome many drawbacks and concerns with conventional cancer treatment methods [6-8]. Metallic nanoparticles possess unique physiochemical properties within biological systems. For example, gold nanoparticles (AuNPs) [9-12], silver nanoparticles (AgNPs) [13-15], iron oxide nanoparticles (Fe_3_O_4_ NPs) [16-18], zinc oxide nanoparticles (ZnO NPs) [19-21], titanium oxide nanoparticles (TiO_2_ NPs) [22-24], etc. have been investigated for many medical therapies. Among all the existing metal nanoparticles, AuNPs possess excellent biocompatibility, surface modification properties, renal clearance, and tissue penetration capabilities [25,26]. AuNPs can be loaded with various drugs via easy surface modifications for enhanced drug delivery and treatments [27,28]. The ability of AuNPs to selectively accumulate in tumor tissues through the enhanced permeation and retention effect (EPR) makes them a better candidate for cancer treatment [29,30]. The optical properties of Au can be fine-tuned by controlling the size and shape of AuNPs to shift from the visible to the near-infrared (NIR) region for deep tissue therapies. The excellent light-absorbing property of AuNPs finds application in photodynamic therapies (PDTs) and the photothermal ablation used for photothermal (PTT) cancer therapies [29,31-33]. It has been reported that the cytotoxicity of AuNPs induced inside cancer cells is nine times more than the effect they create in normal cells. PDT and PTT find reduced side effects with enhanced tumor suppression compared to the conventional methods, but more advancements and modifications are still required for clinical trials. A schematic demonstration of the use of AuNPs in nanobiomedicine is given in Figure 1. By using different synthetic methods and stabilizing agents, the size, shape, and optical properties of AuNPs can be tuned for future biomedical applications. Several approaches are made for inducing cytotoxicity in tumor cells by AuNPs. However, the cytotoxic mechanistic pathways are still unclear, and more investigation is needed in nanomedicine for developing AuNP-based anti-cancer drugs [34,35].

## 2. Gold Nanoparticles in Clinical Practice

The transition of gold nanoconstructs to clinical trials is limited despite the abundant literature on innovative therapeutic formulations involving AuNP. This gap between preclinical and clinical progress is due to a focus on materials-science-driven research, prioritizing multifunctional NPs over clinical efficacy. Successful NP formulations in clinical settings are typically simple, enabling predictable behavior and scalable production. However, their incomplete excretion in vivo and potential long-term side effects present challenges. Table 1 represents a list of gold nanoconstructs which are presently in clinical trials.

## 3. Cancer Immunotherapy

In the past few years, cancer immunotherapy has become an emerging therapy mode for various forms of cancer [37-39]. Various reports and studies showed that patients who underwent cancer immunotherapy showed a longer survival rate and prolonged prognosis than patients treated with chemo- and radiotherapy. Cancer immunotherapy is based on the activation of the native immune system in the human body by a series of pathways through the secretion of cytokines, antibodies, immunity-boosting adjuvants, and tumor vaccines, which attack the abnormal cells [40]. Due to unwanted side effects, the cost, and the lack of target specificity, the application of immunotherapy is limited [41,42]. Various immunotherapeutic agents can be conjugated with metal nanoparticles, especially with AuNPs, for a new treatment modality called nano-immunotherapy [43]. Studies have indicated that nano-immunotherapy has the potential to bolster the effectiveness of immune-based medications against cancer, leading to improved survival rates among numerous cancer patients [44,45].

Nano-immunotherapy enables the delivery of various immune-responsive drugs and cargo into tumor-specific sites. Due to the large surface-to-volume ratio, nanoparticles (NPs) can retain a high drug-loading efficacy. Surface-modified NPs also impart stability and a prolonged circulation time for immune-responsive drugs in the bloodstream. The vital role of NPs is to regulate the native immune system, or, through a specific mode of action, they can reprogram the fate of the cancer cell to be an immune response. From existing literature reports, NPs can be used as a carrier for various immune vaccines, antibodies, and adjuvants, and, specifically, they can target antigen-presenting cells (APCs) or dendritic cells (DCs). NPs can impart the secretion of various cytokines and adoptive cell therapies. They can reprogram the tumor microenvironment (TME) while simultaneously suppressing the tumor response and activating the immune-responsive systems. Moreover, they increase the stability of immune adjuvants or antibodies they carry. Some NPs have an intrinsic adjuvant property that allows them to be used directly for cancer immunotherapy [46].

**Figure 2.**
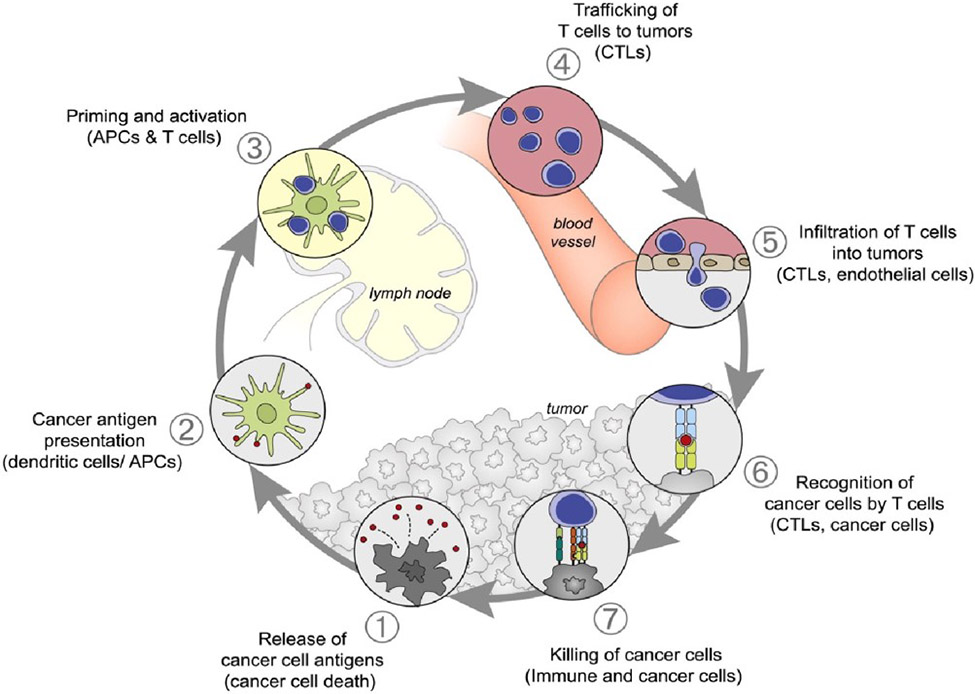
A schematic representation of the cancer immunity cycle. Reproduced with permission from [49]. Copyright 2013, Elsevier.

For an effective anti-cancer immune response, a series of sequential steps known as the cancer-immunity cycle must occur (Figure 2). Initially, neoantigens resulting from cancer development are captured by DCs (step 1). This step requires immunogenic signals, potentially by proinflammatory cytokines from dying tumor cells or the gut microbiota. DCs then present these antigens to T cells (step 2), activating effector T cells against cancerspecific antigens (step 3). The balance of effector T cells to regulatory T cells is crucial at this stage. Activated effector T cells migrate to the tumor site (step 4) and induce the infiltration of T cells into the tumor cells (step 5). The infiltrated T cells then recognize and bind to cancer cells (step 6), ultimately eliminating them (step 7). This process releases more tumor-associated antigens, amplifying the response in subsequent cycles. However, various factors can hinder this cycle’s optimal performance in cancer patients, such as undetected tumor antigens, immune tolerance, impaired T-cell homing, or inhibition within the tumor microenvironment.

Cancer immunotherapy aims to initiate or restart a self-sustaining process of enhancing the body’s immunity against cancer, allowing it to strengthen and spread, but not to the extent that it triggers uncontrolled autoimmune inflammatory responses. Consequently, cancer immunotherapies must be carefully designed to overcome inhibitory mechanisms. While various checkpoints and inhibitors are naturally present in each phase to counteract excessive amplification and can slow down or halt the immune response against tumors, the most effective strategies specifically target the rate-limiting step tailored to each patient’s condition. While boosting the entire cycle may yield anti-cancer benefits, it could also risk causing unintended harm to normal cells and tissues. Recent clinical findings suggest that a common bottleneck in this process is the immune-STAT function, which involves immunosuppression within the tumor microenvironment [47,48]. In cancer patients, these immunity cycles will not work properly, so the activation of DC and T cells is required to boost the immunity cycle, which can be achieved by using AuNPs to deliver antigens, antibodies, adjuvants, vaccines, and immune checkpoint inhibitors [49,50].

This review highlights the advantages of cancer immunotherapy over conventional therapy methods and the use of AuNPs as carriers or direct immunomodulators in cancer immunotherapy. Several studies showed that the unique and favorable characteristics of AuNPs, including the size, shape, charge, biocompatibility, and surface robustness, allow using these NPs as an immunomarker for proteins, genes, and oligonucleotides [45,51]. Recently, a combination of immunotherapy with PTT has also been investigated using AuNPs with different morphologies [52,53].

## 4. Application of AuNPs in Cancer Immunotherapy

### 4.1. AuNPs as Delivery Vehicle for Antigens and Adjuvants

APCs play a pivotal role in bridging the gap between the innate and reprogrammed adaptive immune responses within the cancer immunity cycle. They achieve this by engaging with T cells. APCs induce an adaptive immune response by presenting tumorigenic antigens on their surface via the major histocompatibility complex (MHC) to T cells. However, these antigens often lose their immunogenicity due to various enzymatic processes, preventing them from effectively entering APCs. AuNPs, known for their specificity and stability, offer a solution by delivering antigens directly to APCs, bypassing enzymatic degradation, and amplifying both T-cell and DC immune responses.

Batus et al. demonstrated that 10 nm-sized AuNPs linked to peptides triggered the activation of murine-bone-marrow-derived macrophages, leading to the induction of proinflammatory factors, such as tumor necrosis factor (TNF), interleukin IL-1β, IL-6, and nitric oxide (NO) [54]. Their research revealed that, while both individual AuNPs and peptideinduced macrophages showed an increased proliferation, the nano-conjugates suppressed the macrophage proliferation rate. Furthermore, their findings emphasized that the specific peptide pattern on the nanoparticles was pivotal in influencing the proliferation rate, surpassing the significance of the peptide length and polarity. In a separate investigation, Fallarini et al. functionalized gold nanoparticles with an average size ranging between 2–5 nm with disaccharides. These modified nanoparticles prompted macrophage activation, T-cell proliferation, and elevated IL-2 production [55]. A more detailed examination revealed that 5 nm-sized AuNPs bearing disaccharides were more effective in stimulating T cells than AuNPs with monosaccharides, which is attributed to the degradation of AuNPs at this stage.

AuNPs, through the appropriate surface modifications, can traverse blood vessels and biological barriers, facilitating a targeted delivery to stimulate T cells and DCs. Shinchi et al. showcased the potential of utilizing gold nanoparticles (AuNPs) adorned with αmannose as vehicles for delivering a synthetic toll-like receptor (TLR7) ligand, 2-methoxy ethoxy-8-oxo-9-(4-carboxy benzyl) adenine (1V209), through comprehensive in vitro and in vivo investigations [56]. Their methodology entailed binding α-mannose and the TLR7 ligand onto AuNPs’ surface utilizing thioctic acid. In vitro experiments on mouse bone marrow demonstrated a heightened cytokine production in dendritic cells (DCs) and human peripheral blood mononuclear cells compared to untreated samples. In vivo studies unveiled that the application of 1V209 nano-conjugates notably boosted the levels of IgG2c antibodies specific to ovalbumin, surpassing those elicited by unconjugated materials. This groundbreaking strategy sets the stage for integrating antigens and adjuvants into AuNPs, paving the way for advanced immunotherapy modalities.

Polysaccharide-coated AuNPs exhibit remarkable efficacy in cancer immunotherapy by activating DCs and T cells. In a study conducted by Zhang et al., they found that AuNPs coated with polysaccharides from Ganoderma lucidum, measuring an average size of 20.8 ± 2.2 nm, led to elevated levels of CD86^+^, CD80^+^, CD40^+^, and MHCII expression. Additionally, these nanoparticles stimulated the proliferation of CD4^+^ and CD8^+^ T cells within the 4T1 cell line. This research suggests that the integration of immune-active polysaccharides into AuNPs can enhance their circulation time and stability, potentially reducing the reliance on chemotherapeutic agents in cancer treatment [57].

Cytosine-phosphate-guanine (CpG) is a potent natural stimulant for the immune system. It interacts with toll-like receptors found in APCs, triggering the release of cytokines and programming CD8^+^ T cells. When combined with cancer drugs, CpG adjuvants, AuNPs exhibit remarkable macrophage-clearing capabilities, potentiating and enhancing immune responses in both T cells and DCs. In their study, Lin et al. demonstrated that incorporating oligonucleotide-modified CpG into AuNPs can facilitate CpG delivery without any adverse effects. Notably, nanoparticles smaller than 15 nm exhibited exceptional immune-boosting effects by activating CpG-stimulated macrophages. Furthermore, in vivo experiments conducted in mice revealed that these CpG-AuNPs can effectively inhibit tumor growth and enhance the survival rate of the mice. Importantly, AuNPs also contribute to maintaining the stability of the CpG adjuvant throughout the tumor cycle without compromising the integrity of the DNA tag [58].

**Figure 3.**
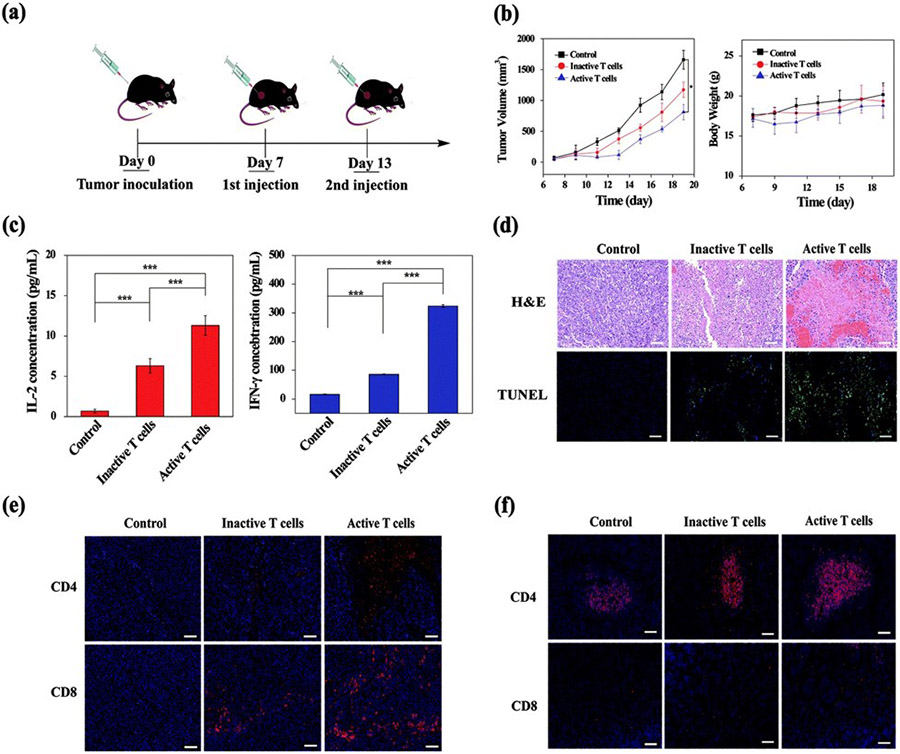
Illustrates various aspects of the experimental procedure and outcomes. (**a**) outlines the treatment protocol involving intravenous injection in vivo. (**b**) depicts the progression of tumor growth alongside changes in body weight across different experimental groups of mice over time. (**c**) showcases the results of ELISA tests conducted on mouse serum to measure levels of IFN-γ and IL-2. (**d**) presents histological images of tumor sections stained with H&E and TUNEL, illustrating differences between mouse groups. (**e,f**) display representative immunofluorescence staining of tumor and spleen sections, respectively, highlighting the expression of CD3, CD4, and CD8 markers, with DAPI staining indicating tumor cell nuclei. Scale bar = 100 μm. *** *p* < 0.001. Reproduced with permission from [59]. Copyright 2020, Royal Society of Chemistry.

Dendrimer-encapsulated AuNPs present a promising avenue for efficient CpG vector delivery into DCs. In their research, Chen et al. utilized poly(amidoamine) (PAMAM)AuNP nanoconjugates to transport CpG into bone-marrow-derived dendritic cells (Figure 3a,b) [59]. The challenge of delivering CpG, with its negative surface charge, into DCs was addressed by employing positively charged PAMAM dendrimers to encapsulate both CpG and AuNPs, thereby enhancing the biocompatibility and transfection efficiency of the conjugate (Figure 3c,d). Both in vitro and in vivo experiments using xenografted melanoma tumor models demonstrated that this nanoconjugate could effectively enhance and activate T cells within BMDCs, leading to an improved CD4^+^ and CD8^+^ antigen expression following intravenous injection as opposed to intratumoral injection (Figure 3e,f). A similar approach was adopted for transporting CpG-oligodeoxy nucleotides to enhance the antigen expression in BMDCs and activate T cells [60]. To augment the antigen-loading efficiency onto dendrimer-coated AuNPs, zwitterionic 2-methacryloyloxyethyl phosphorylcholine was employed, resulting in a substantial increase in CpG-oligodeoxy nucleotide loading and T-cell maturation. Furthermore, Luo et al. demonstrated that thiolate CpGoligonucleotide-bearing hollow AuNPs exhibited a remarkable 15-fold enhancement in TNF-α secretion from RAW264.7 cells compared to CpG or CpG-oligonucleotide alone, underscoring the potential utility of hollow AuNPs for cancer immunotherapy [61]. In another study, Yue et al. showed that CpG-coated AuNPs of different sizes and shapes responded differently to the activation of toll-like receptor 9 [62]. Spherical AuNPs with a 13 nm size showed a high specificity for the toll-like receptor 9 activation, whereas 50 nmand 40 nm-sized Au nanostars showed a higher cellular uptake and immune activation due to their off-target effects.

Michelini et al. investigated how the size of AuNPs affects the maturation of DCs and their response to T-cell activation [63]. They utilized AuNPs loaded with lipopolysaccharide (O-antigen) to examine their ability to induce maturation in DCs. The findings revealed that, without O-antigen, DCs did not exhibit a response. Different sizes of AuNPs (4, 8, 11, and 26 nm) were employed in the study, with the 26 nm-sized AuNPs showing a notably high activation rate for DCs. This activation was characterized by the downregulation of CD86^+^, IL-12, and IL-27, the upregulation of ILT3, and the formation of E-class compartments. While further investigations are needed to understand the mechanism fully, this study underscored that AuNPs are safe for T-cell activation under homeostatic conditions.

Alternatively, AuNPs present a promising avenue for cancer immunotherapy through the functionalization with cytokine-type TNF. In a study by Paciotti et al., thiolated PEGmodified AuNPs, sized at an average of 33 nm, effectively delivered recombinant human TNF to MC-38 colon carcinoma tumors. This approach yielded a notably enhanced antitumor response compared to TNF administered alone while mitigating its toxicity [64,65]. Moreover, AuNPs offer a platform for combined antigen and adjuvant delivery to promote dendritic cell maturation and adoptive immunotherapy. Zhou et al. demonstrated this potential by developing a nano-Au-cocktail integrating two distinct AuNP systems, one featuring a CpG adjuvant and the other an ovalbumin peptide antigen [66]. Their findings highlighted the in vivo lymphoid tissue homing and activation of CD8^+^ T-cell responses following intravenous administration of the nano-Au-cocktail. Collectively, these studies underscore the capacity of AuNPs to serve as effective carriers for antigens and adjuvants in cancer immunotherapy. By modulating the surface properties and chemistry, AuNPs offer a versatile and biocompatible platform for advancing immunotherapeutic strategies against cancer.

### 4.2. AuNPs as Delivery Vehicle for Cancer Vaccines

Cancer vaccines present a potential avenue for cancer immunotherapy [67-69]. However, their effectiveness in stimulating immune responses and T-cell activation is limited. Additionally, there are challenges associated with the target-specificity within tumors and delivering sufficient vaccine doses [70]. Numerous studies indicate that AuNPs offer a promising solution to address these limitations of cancer vaccines. AuNPs can increase the vaccine payload and improve their bioavailability at tumor sites, primarily because of their remarkable size adjustability.

Arnaiz et al. presented a groundbreaking study in the realm of HIV research, unveiling the potential of AuNPs as a tool against the virus [71]. With an average size of 1.8 nm and engineered with the HIV gp120 antigen, these AuNPs exhibited a remarkable resemblance to oligomannosides, crucial components in the HIV infection process. This biomimicry triggered the maturation of DCs, hindering HIV infection by stimulating T cells through endocytosis. The AuNPs showcased the precise targeting of DCs by binding to C-type lectin receptors and the n-terminal mannose glycans on HIV gp120, facilitated by intricate calcium interactions. This study not only sheds light on the potential of mannose-coated AuNPs as cancer vaccines but also emphasizes their role in specific DC maturation and T-cell immunotherapy, offering promising avenues for future research and therapeutic interventions.

In a study led by Lin et al., gold nanoparticles (AuNPs) effectively transported tumortargeting antigen peptides to tumor sites [72]. By employing carbodiimide chemistry, they incorporated three different MHC class I peptides, including those from the antigen model OVA and melanoma antigens gp100 and Trp-2, into 30 nm-sized AuNPs. This method achieved an impressive 90% peptide-loading efficiency. The resulting AuNP vaccine demonstrated minimal cytotoxicity while significantly enhancing the stimulation of cytotoxic T lymphocytes, outperforming free antigen peptide vaccines by fourfold. An evaluation of anti-tumor immunogenicity showed the highest efficacy with AuNP vaccines containing ovalbumin and gp100 antigen peptides. Additionally, Trabbie et al. investigated the use of polysaccharide-coated AuNPs to target APCs expressing Dectin-1 [73]. Their research indicated that AuNPs coated with polysaccharide β-1,3-glucans elicited an in vivo immune response, activating antigen-recognizing T immune cells. Furthermore, this approach maintained a balanced expression of cytokines conducive to anti-tumor immunity, with a limited expression of the immunosuppressive cytokine, Il-10.

Cancer vaccines and their formulations primarily aim to stimulate the formation of MHC type I complexes, crucial for eliciting CD8^+^ cytotoxic T-cell responses. In a groundbreaking study by Cao et al., a new strategy for developing an anti-cancer vaccine was introduced [74]. They ingeniously employed 50 nm-sized AuNPs coated with thiolated hyaluronic acid and ovalbumin antigen. These nanoparticles capitalized on their photothermal properties upon near-infrared light exposure, enabling the precise delivery of the antigen into the cytosol. By utilizing laser irradiation, this process induced the disruption of endo-/lysosomes, heightened the production of reactive oxygen species, and increased proteasome activity and the consequent MHC I antigen presentation. Ultimately, this innovative approach effectively spurred the activation of CD8^+^ T cells, fostering a potent anti-cancer immune response.

Recent years have seen a proliferation of instances where a combination of cancer adjuvants and cancer vaccines, employing AuNPs, have demonstrated numerous successful outcomes [75,76]. This dual-delivery approach not only triggers a targeted antibody response against tumor cells but also activates antigen-specific cytotoxic lymphocyte antigen responses. One notable example involves MUC1, a glycoprotein commonly overexpressed in various tumor tissues. Liu et al. developed an anti-tumor vaccine featuring MUC1 and α-GalCer, with AuNPs as the delivery vehicle [77]. Studies have demonstrated that this nanoparticle-based vaccine approach significantly enhances tumor suppression in mouse models. In another study, Lee et al. devised a nanocomplex adjuvant vaccine utilizing 7 nm-sized AuNPs to stimulate T-cell responses in lung metastatic studies, employing B16F10 melanoma tumor-bearing mice [78]. They used red fluorescent protein as the antigen and CpG 1668 as the adjuvant. Following injection, the nano-vaccine accumulated in lymph nodes, interacted with DCs, and elicited a T-cell immune response through the Th1 pathway.

The literature presented suggests that AuNPs have the potential to serve as carriers for tumor vaccines, offering an improved payload capacity and specificity. Furthermore, AuNPs have been observed to instigate substantial alterations in T-cell responses, assuming a crucial role in cancer immunotherapy. Consequently, AuNP-based cancer vaccines emerge as a promising and innovative avenue, poised for application in future immunotherapeutic contexts.

### 4.3. AuNPs as a Delivery Vehicle for Antibodies

Various antibody-based medications, including nivolumab, pembrolizumab, cemiplimab, atezolizumab, durvalumab, and avelumab, are integral to cancer immunotherapy research [79]. Despite their potential, challenges such as the cost, diminished clinical efficacy, side effects, and substantial loading requirements persist, hindering their widespread use. Many cancer immunotherapies focus on activating immune checkpoints like programmed cell death protein (PD-1) and programmed cell death ligand (PDL-1) to bolster the body’s natural defense against cancer cells [80]. Presently, AuNPs are revolutionizing antibody delivery to tumor sites, owing to their customizable surface modifications. AuNPs offer precise antibody targeting capabilities due to their surface functionalization, and their high surface-to-volume ratio enhances the antibody loading efficiency. PD-1, predominantly found in T cells, stands as a key immune checkpoint frequently targeted in cancer therapy. Its interaction with overexpressed PDL-1 ligands in tumor cells dampens T-cell immune responsiveness, leading to immune suppression and tumor growth inhibition [81,82].

AuNPs, offer promising solutions to a range of challenges in cancer treatment, including drug resistance and the targeted delivery of therapeutic agents like PDL-1 antibodies to tumor sites. Their unique surface properties enable the efficient traversal of the tumor microenvironment barrier when coupled with antibodies and drugs. In a study by Emami et al., AuNPs synthesized and linked to doxorubicin and anti-PDL-1 antibodies using lipoic acid polyethylene glycol exhibited an average size of 40 nm [83]. Through NIR irradiation experiments, a notable 66% apoptosis rate was observed in the CT-26 cell line, showcasing the efficacy of this novel drug delivery system for colorectal cancer treatment.

AuNPs, due to their high X-ray attenuation ability, are valuable as contrast agents in computed tomography (CT) imaging. The fusion of immune checkpoint inhibitors with AuNPs offers exciting prospects for a novel theranostic approach in cancer immunotherapy. Meir et al.’s research demonstrated that AuNPs linked with anti-PDL-1 antibodies induced a potent anti-tumor response while needing minimal immune checkpoint ligands [84]. Moreover, CT scans of mice injected with these AuNPs showed a direct correlation between tumor growth and T-cell infiltration, suggesting the potential use of this engineered AuNP system to evaluate treatment efficacy by noninvasive imaging. In a broader perspective, AuNPs hold promise for enhancing the targeted delivery of various antibodies to suppress PDL-1 expression. However, thorough clinical trials are imperative to gather comprehensive insights, as many studies have remained confined to in vitro environments.

### 4.4. AuNPs as Delivery Vehicle for Genetic Drugs

The advent of genetic drugs like siRNA and their nanoformulations has revolutionized the synthesis of diverse antigens and the stimulation of immune responses. These groundbreaking techniques hold immense promise in cancer immunotherapy [85]. Among the arsenal of tools available, AuNPs stand out for their remarkable efficiency in loading DNA/RNA via compatible functional groups. SiRNA can be seamlessly incorporated into AuNPs, presenting a robust solution for maintaining siRNA stability under physiological conditions—a feat that is often challenging [86,87]. Moreover, AuNPs offer lower toxicity than other nanoparticle variants, facilitating selective gene manipulation and siRNA transfection with enhanced safety profiles [43,44,85].

Hou et al. conducted a study aimed at developing a novel nanocomplex incorporating PAMAM-dendrimer-encased AuNPs, partially modified with polyethylene glycol monomethyl ether [88]. Their focus was on enhancing cancer immunotherapy by conjugating pDNA/siRNA, specifically targeting B-cell lymphoma 2 in human cervical cancer cells. Their findings demonstrated the successful inhibition of green fluorescent protein and luciferase reporter gene expression by the nanocarrier. Concurrently, Labala et al. investigated a layer-by-layer assembly method combining AuNPs with anti-STAT3 siRNA or imatinib mesylate for melanoma treatment [89]. Their experiments on B16F10 melanoma cells showcased the potential of both STAT3 siRNA-loaded and imatinib mesylate-loaded AuNPs to induce apoptosis and decrease cell viability independently. Notably, co-delivering these therapeutic agents via AuNPs’ layer-by-layer assembly resulted in a substantial reduction in tumor volume and weight, along with a significant suppression of STAT3 protein expression. Consequently, this co-delivery approach utilizing AuNPs as nanocarriers holds promise for future studies in immunotherapy and topical iontophoretic applications.

In a study by Xue et al., they developed PAMAM-dendrimer-coated AuNPs aimed at delivering PD-L1 small interfering RNA (siPD-L1) to enhance cancer immunotherapy [90]. The siPD-L1 effectively inhibited PD-L1 gene expression, while the AuNPs ensured stability, biocompatibility, and the effective gene delivery to tumor sites. This SiRNA-AuNPs combination showed promising immunotherapeutic potential, as indicated by the increased infiltration of CD8^+^ and CD4^+^ T cells in both spleen and cancerous tissue, suggesting T-cell activation against tumor cells. Furthermore, Gulla et al. demonstrated in another study that mice with melanoma showed a significant survival rate exceeding 75% when treated with AuNPs-CGKRK peptide (ranging from 17 to 80 nm) in combination with SiRNA-PDL-1 and STAT3 SiRNA genes, outperforming individual AuNPs and genes used [91].

**Figure 4.**
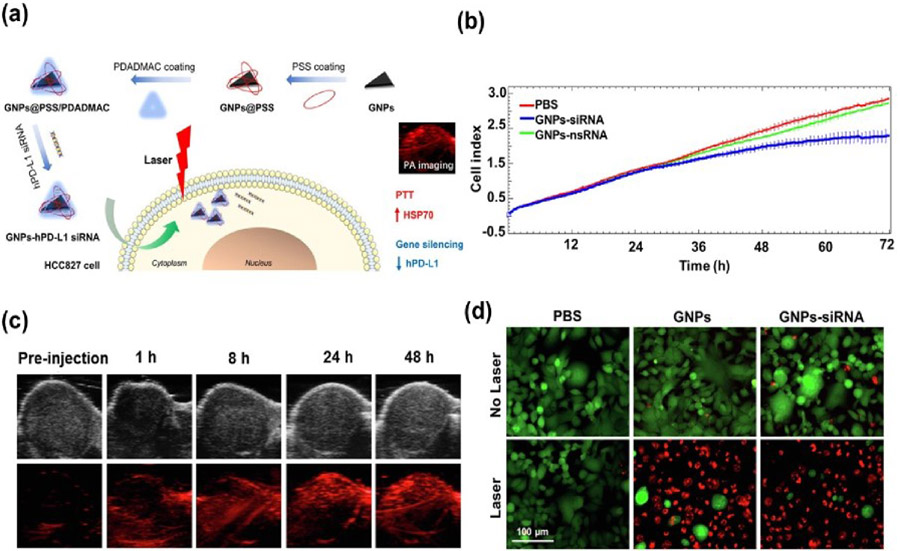
(**a**) Illustration of Au nanoprism-based nanocarrier for siRNA-PDL-1 complex in cancer immunotherapy. (**b**) Impact of hPD-L1 gene silencing on HCC827 cell growth after treatment with PBS, GNPs-siRNA, or GNPs-nsRNA. (**c**) Sequential photoacoustic imaging pre- and post-injection of GNPs-hPD-L1 siRNA at 1, 8, 24, and 48 h using a 650 nm laser. (**d**) Fluorescence images of Calcein AM/PI-stained HCC827 cells incubated with PBS, GNPs, and GNPs-siRNA post-laser irradiation (3 min, 0.8 W cm^−2^) for 24 h. Reproduced with permission from [92]. Copyright, 2019, Elsevier.

Liu and colleagues developed a method for loading siRNA-PDL-1 complexes into gold nanoprisms (GNPs) for precise delivery to lung cancer sites (Figure 4a) [92]. To enhance the adsorption of negatively charged siRNA, the GNPs’ surface was coated with poly(sodium 4-styrenesulfonate) (PSS) and poly(diallyldimethylammonium) chloride (PDADMA). Both in vitro and in vivo experiments confirmed the effective delivery of the siRNA-PDL-1 complex by this synthesized nanocarrier, leading to the suppression of hPD-PDL-1 expression in HCC827 cells (Figure 4b). Additionally, the resulting nanocomplex, leveraging the optical properties of GNPs, was utilized for photoacoustic imaging and photothermal therapy studies for lung cancer (Figure 4c,d). A photoacoustic tomography analysis revealed a progressive increase in photoacoustic signals within tumor cells one hour post-injection, maintaining intensity levels for up to 48 h, demonstrating the remarkable tumor-targeting ability and intratumoral distribution of GNPs-hPD-L1 siRNA in vivo. The evaluation of therapeutic efficacy using a 633 nm laser source (3 min, 0.8 W cm^−2^) indicated a reduced cell viability in GNPs-hPD-L1 siRNA-treated HCC827 cells through confocal microscopic studies with propidium iodide (Figure 4d). Furthermore, the therapeutic efficiency with GNPs-hPD-L1 siRNA was compared to that without nanoparticles, as evidenced by a flow cytometry analysis.

Thus, from the existing literature reports, it is clear that AuNPs, along with siRNA oligonucleotides for silencing specific genes, can constitute immunotherapeutic agents. This platform opens the protocol for developing new drug delivery carriers for site-specific tumor immunotherapy.

### 4.5. Role of AuNPs in the Tumor Microenvironment

Tumor growth induces changes in the TME, like acidity, hypoxia, and irregularity in the vascular tissue structures. The TME can generate immune-responsive tumor suppressors, cells, regulators, and cytokine mediators. Studies showed that AuNPs can improve hypoxia conditions and regulate cytokine expressions in the TME. Ibrahim et al. showed that AuNPs with sizes 5, 20, and 50 nm can increase IL-1β and IL-6 expression after one day of injection, and tumor necrosis factor in the liver, kidney, and spleen of mice with a suitable dose of AuNPs [93]. AuNPs controlled the TNF-cytokine expression and improved the proinflammatory response in tumor-bearing mice. The regulated changes in cytokine expression improved the hypoxia state in tumor cells, thereby enhancing the survival rate. This study correlated the size of AuNPs and the interleukin expressions, concluding that AuNPs with an average size of 5 nm improved the mRNA expression in IL-1β and IL-6 2.312 ± 0.737-fold in the liver compared with 20 nm- and 50 nm-sized AuNPs. The studies showed that the administration of AuNPs at regular intervals for two months does not induce any histopathological changes in the kidney, liver, or spleen, indicating the advantage of AuNPs for cancer immunotherapy.

**Figure 5.**
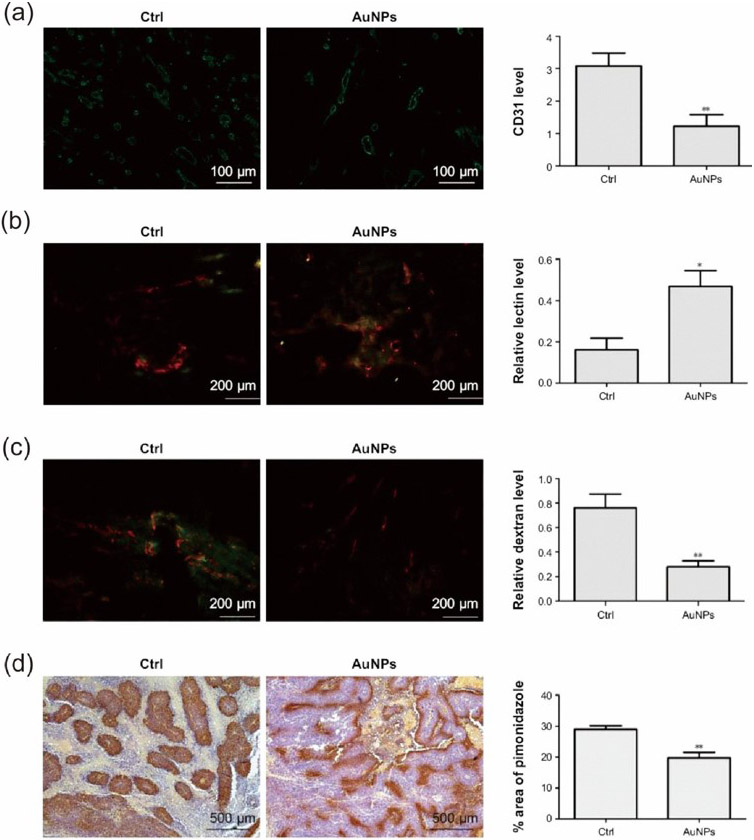
Representing the role of AuNPs for normalizing the tumor vasculature. (**a**) CD31 staining studies. Results showed that, by the administration of AuNPs, the tumor vessel area decreased. (**b**) The improved perfusion rate in the tumor by AuNPs as studied by the FITC-conjugated lectin (green) and CD31 (red) staining. (**c**) The reduced vascular leakage in the tumor vessels by AuNPs as studied by the FITC-dextran (green) and CD31 staining. (**d**) Improved hypoxia condition in tumor cells as studied by the pimonidazole staining after treatment with AuNPs. * *p* < 0.05, ** *p* < 0.01. Reproduced with permission from [95]. Copyright 2017, Dove Medical Press.

Due to the changes in the pathological features of the TME, most immunotherapies are inefficient. Many researchers are involved in developing immune vaccines that can reprogram or reshape the tumor immunosuppressive microenvironments [94]. The perfusion in tumor vascular vessels creates extreme hypoxia conditions, so controlling the shape and structural features of blood vessels in the tumor is one key factor in controlling immunosuppressive TME. Many studies showed that AuNPs can control the morphology and perfusion, and reduce hypoxia by improving fluid motions in tumor cells [95-97]. Li et al. showed that AuNPs facilitate tumor vasculature normalization and oxygen levels by an angiogenin type I receptor pathway [95]. AuNPs with an average diameter of 15 nm were prepared, and tumor angiogenesis was studied using CD31 endothelial markers for B16F10 cells in mice. After treatment with the AuNPs, the vascular density decreased and possessed a uniform morphology compared to the controls (Figure 5a). A Lectin+CD31 analysis showed an improved vascular perfusion rate (Figure 5b). The dextran leakage studies showed minimal leakage, indicating the maturation of tumor blood vessels (Figure 5c). Further studies showed that AuNPs can decrease the vascular endothelial growth factor (VEGF) in tumor expressions and inhibit the epithelial–mesenchymal transition. Therefore, the reprogrammed immune responsive system inhibits the MMP-2 and c-Myc gene expressions with improved hypoxia conditions (Figure 5d).

Immunotherapy depends on the involvement of DCs and macrophages to induce the innate anti-tumor effects along with the cytokine and inflammatory secretions in the TME. The hypoxia conditions in tumor cells alter their physiological environments, so most anti-tumor vaccines and drugs cannot reach the tumor sites in the TME. AuNPs can target the abnormal components of the TME and reprogram or regulate the immune-responsive nature. Overcheck et al. reported that AuNPs can control the secretion of abnormal VEGF and transforming growth factor β (TGF-β), which are mainly responsible for the inhibition of DCs and macrophages [98].

Another issue in cancer immunotherapy is the angiogenesis suppression by the acquired resistance of the endothelium towards the drugs, especially in anti-VEGF therapies. Zhang et al. showed that AuNPs can disrupt the TME crosstalk and can render or inhibit angiogenesis in vitro [99]. AuNPs disturb the signal transduction between the TME and the endothelial cells and reduce the migration and tube formation in endothelial cells. The mechanistic study showed that AuNPs can deplete almost 95% of the VEGF165 from VEGF, and it could remove 45% of the VEGF165 from the conditioned media. This study showed that AuNPs inhibit angiogenesis via the blockade of VEGF-VEGFR2 and inhibit the signal processing between the TME and endothelial cells by the perturbed crosstalk between them. In another study, Huang et al. showed that tumor vascular normalization and angiogenesis can be retained by using folic-acid-coated AuNPs with target specificity [100]. The polymercoated AuNPs showed tumor proliferation inhibition for both in vitro and in vivo studies with enhanced tumor metastasis suppression for in vivo. The folic-acid-coated AuNPs enhanced the perfusion and alleviated hypoxia by infiltrating CD3^+^CD8^+^ T lymphocytes.

Thus, AuNPs can impart vascular normalization and activate the immunoreponsive TME. In the susceptible microenvironment, they can be further used to secrete cytokines and chemokines for the maturation of DCs and T cells in cancer immunotherapy.

## 5. Cancer Immunotherapy Using AuNPs via PDT and PTT

**Figure 6.**
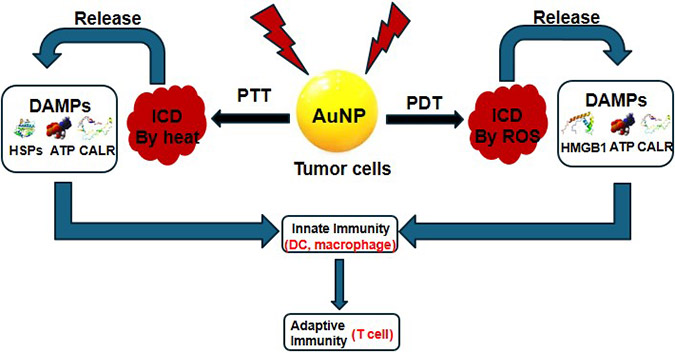
Insights into the mechanistic induction of the host immune system through the application of AuNP-based strategies for cancer treatment, including PDT and PTT.

AuNPs exhibit remarkable photothermal and photosensitization properties by manipulating their size and shape. These unique attributes make AuNPs a strategic tool in anti-tumor medicine, particularly in photothermal and photodynamic therapies [25]. In photothermal therapy (PTT), AuNPs efficiently convert light into heat, enabling their use in tumor ablations. Conversely, in photodynamic therapy (PDT), AuNPs serve as potent photosensitizers, catalyzing the generation of cytotoxic reactive oxygen species (ROS) for tumor suppression. Recent years have witnessed a plethora of literature exploring AuNPs for PTT [33,101] and PDT [102,103], both as standalone treatments and in combination with chemotherapy [104], radiation therapy [105], or immunotherapy [106,107]. In this section, we will shed light on the pivotal role of AuNPs in PTT- and PDT-based cancer immunotherapy. Both PTT and PDT not only induce cancer cell death but also trigger the immune system to release antigens and heat shock proteins (HSPs). These molecules are subsequently recognized by various antigen-presenting cells and tissues, initiating a cascade of immune responses involving DCs and T cells (Figure 6).

### 5.1. Application of AuNPs for PTT-Based Immunotherapy

#### 5.1.1. PTT-Based Immunotherapy for Direct Immunogenic Cell Death

The utilization of AuNPs in PTT demonstrates considerable potential in the treatment of diverse tumor types. However, a significant obstacle in PTT-based approaches is the limited tissue penetration of light. This challenge can be effectively addressed by employing AuNPs, which can absorb light in the near-infrared (NIR-I and NIR-II) regions. Consequently, PTT can induce the production of damage-associated molecular patterns (DAMPs), initiating various immune response pathways crucial for tumor treatment.

**Figure 7.**
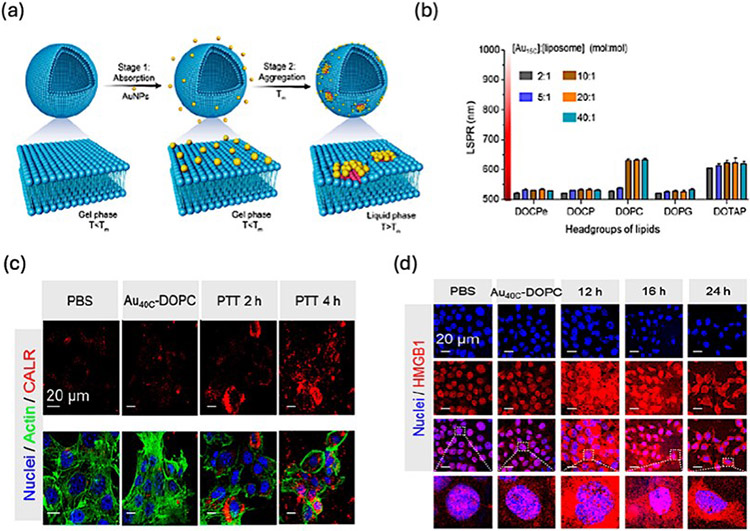
(**a**) Schematic depiction illustrating the dynamic self-assembly process of AuNPs within liposomes. (**b**) Evolution of absorption peak shifts in AuNPs as influenced by varying compositions of AuNPs and liposomes. (**c**) Confocal microscopy images showcasing Calreticulin (CALR) expression in 4T1 tumor cell post-photothermal therapy (PTT) treatment. (**d**) Immunofluorescence staining analysis revealing the expression levels of HMGB1 in cancer cells following PTT intervention. Reproduced with permission from [108]. Copyright 2019, American Chemical Society.

In a specific investigation, Ma et al. showcased that incorporating AuNPs into liposomes led to a notable shift in their absorption properties towards the NIR II region due to self-assembly, as depicted in Figure 7a [108]. Through the meticulous optimization of both AuNP size and liposome composition, they achieved a significant redshift of the absorption peak to NIR-II at 964 nm for Au40C-DOPC, resulting in an impressive photothermal conversion efficiency of 21.88%, as shown in Figure 7b. In vitro experiments revealed that PTT induced immunogenic cell death (ICD), prompting the production of DAMPs. Conversely, in vivo investigations demonstrated a more consistent generation and distribution of DAMPs in the NIR-II region during effective PTT for tumor therapy, eliciting both innate and adaptive immune responses, as illustrated in Figure 7c,d. NIR-II laser irradiation facilitated the generation of IFNγ-producing CD4^+^/CD8^+^ T cells and natural killer (NK) cells through DC maturation. The utilization of the self-assembly method of AuNPs emerged as a potent strategy for cancer immunotherapy using PTT, even applicable for distal and metastatic cancers.

#### 5.1.2. PTT Combined with Immunoadjuvants

**Figure 8.**
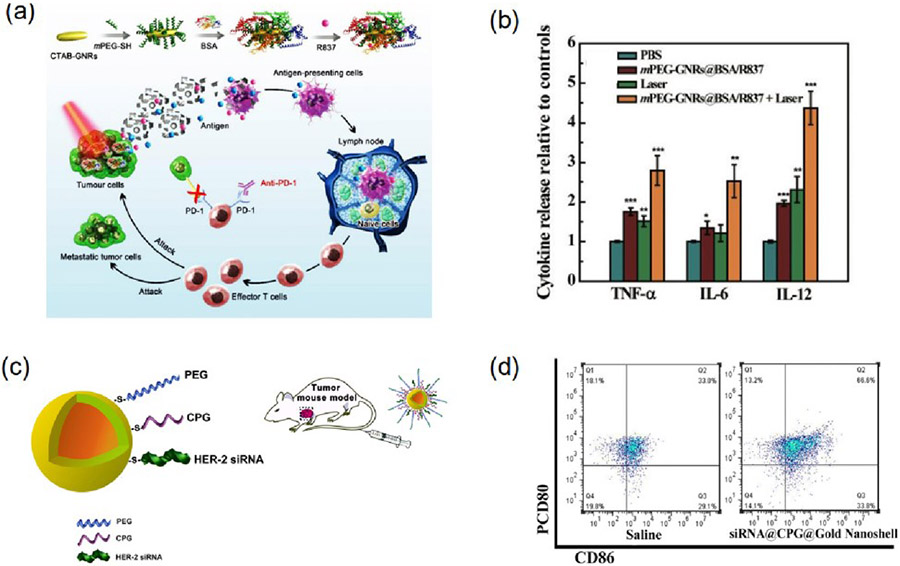
Role of AuNPs in PTT based immunotherapy using immunoadjuvants. (**a**) A schematic for mPEG-GNRs@BSA/R837 nanocomplexes synthesizes and elucidates the mechanism underlying their stimulation of anti-tumor immune responses. (**b**) TNF-α, IL-6, and IL-12 cytokines levels measured in the serum of mice after three days of laser treatment. (**c**) A schematic representation of gold Nanoshell drug delivery system. (**d**) Induction of dendritic cell maturation through siRNA@CPG@Gold Nanoshell-mediated photothermal therapy in mice with MFC tumors. Cells from the lymph nodes draining the tumors were harvested 72 h post-treatment and analyzed via flow cytometry after staining for CD80 and CD86. Where * *p* < 0.05, ** *p* < 0.01 and *** *p* < 0.001. Reproduced with permission from [109,110]. Copyright 2018, Royal Society of Chemistry. Copyright 2019, Springer.

Photothermal therapy utilizing immunoadjuvants represents a promising avenue for cancer immunotherapy. By coupling diverse immunoadjuvants with AuNPs, DC maturation and T-cell activation within tumor cells have been induced. Notably, the photothermal efficiency of Au nanorods surpasses that of spherical AuNPs. In a study by Zhou et al., biologically inspired bovine serum albumin (BSA)-coated Au nanorods, boasting an average diameter of 122.1 ± 11.6 nm, were engineered [109]. These nanorods were loaded with cetyltrimethylammonium bromide and the immunoadjuvant imiquimod (R837), known for eliciting robust immune responses (Figure 8a). The photothermal therapy triggered the secretion of various immune-responsive cytokines such as TNF-α, IL-6, and IL-12 (Figure 8b). Combining the immunoadjuvant with photothermal therapy induced by AuNRs resulted in a remarkable 65.1% inhibition of tumor growth, concurrently activating T cells and dendritic cells, thus bolstering the memory immune response.

Studies have demonstrated that Au nanoshells possess exceptional photothermal conversion efficiencies and adjuvant loading capacities. For instance, Zhang et al. engineered an Au nanoshell delivery system incorporating siRNA and CpG adjuvant, revealing a remarkable tumor ablation efficacy and eliciting an immune response against gastric tumor cells [110] (Figure 8c). The nanomaterials were effectively distributed among cancer cells and demonstrated a staggering 66.9% induction of apoptosis within just 5 min of laser irradiation, leading to prolonged survival rates in tumor-bearing mice. Additionally, flow cytometric analyses indicated that the nanomaterial system induced dendritic cell maturation by up to 66%, accompanied by elevated levels of IL-2 and IL-6 (Figure 8d). In another investigation, Chen et al. demonstrated the efficacy of polyethyleneimineprotected Au nanorods complexed with CpG adjuvant, exhibiting potent photothermal and immunotherapy responses against 4T1 cells [111]. Laser irradiation studies revealed a significant increase in both early and late apoptotic cells 28.4% and 70.4%, respectively, along with a notable dendritic cell infiltration. Furthermore, Yata et al. developed an immunostimulatory hydrogel utilizing AuNPs with CpG adjuvant and DNA, resulting in the secretion of anti-tumor inflammatory mediators (TNF-α, IL-6, and IFN-γ) and enhanced survival rates in mice with efficient tumor ablation [112]. These findings underscore the potential of this nanoparticle system for combined photothermal therapy and immunotherapy investigations.

#### 5.1.3. PTT Combined with Immune Checkpoint Inhibitors

Cancer immunotherapy has seen significant advancements through the targeted inhibition of specific immune checkpoints, particularly cytotoxic T lymphocyte antigen-4 (CTLA-4) and PD-1/PD-L1. Blocking these checkpoints holds promise for enhancing cancer immunotherapy by activating T cells and reducing immune suppression within the TME. Studies have demonstrated that combining AuNPs with PD-1/PD-L1 immune checkpoint inhibitors is an effective strategy for photothermal-therapy-induced immune responses [113].

Liu et al. demonstrated that Au nanostars exhibit a robust photothermal therapy (PTT) response, and, when combined with the PD-L1 immune checkpoint blockade, they induce highly effective immune photothermal therapy for MB49 bladder cancer cells in mice [114]. Their study revealed that mice with tumors exhibited significantly elevated T cells, CD4^+^ T cells, CD8^+^ T cells, and B cells in the spleen seven days post-treatment. In contrast, the population of myeloid-derived suppressor cells was markedly reduced. Furthermore, the combined immune therapy led to the upregulation of PD-1 expression on both CD4^+^ and CD8^+^ T cells, resulting in increased proportions of PD-1+CD4^+^ and PD-1+CD8^+^ T cells, ultimately improving the survival rate of the mice. In a separate investigation, Odion et al. demonstrated that Au nanostars could stimulate the release of tumor-associated antigens, heat shock proteins, and DAMPs [115]. Combining photothermal irradiation with anti-PDL-1 checkpoint inhibitors significantly enhanced the survival rate in a cohort of mice harboring tumor cells.

Yang et al. demonstrated the efficacy of an Au@Pt nanoparticle system combined with a strategically designed peptide conjugate for inducing potent cancer photothermal immunotherapy [116]. Their peptide design facilitated the production of a D-peptide antagonist targeting PD-L1 during PTT. In vivo studies conducted on 4T1 breast cancer models revealed an enhanced T-cell generation through the PD-L1 immune checkpoint blockade, resulting in the eradication of primary tumors and suppression of lung metastasis. Similarly, Cheng et al. illustrated the versatility of Au nanocages as a multifunctional nanomaterial for cancer immunotherapy [117]. By loading Au nanocages with Ansamitocin P3 (AP3) and anti-PDL1 binding (AP3-Au-anti-PDL1), they achieved an excellent immune response upon irradiation with an NIR light for 10 min. This approach stimulated highly activated DCs through a controlled AP3 release, enhancing T-cell proliferation, particularly in treating hepatocellular carcinoma. Additionally, Luo et al. presented promising results with hollow Au nanoshells coated with Poly (d, l-lactic-co-glycolide) loaded with anti-PD-1 peptide for cancer immunotherapy using an NIR laser [118]. This nanocarrier system effectively eliminated primary tumors and inhibited the growth of distant uninfected primary tumors, resulting in prolonged survival in mice by promoting CD8^+^ T-cell generation. Notably, elevated levels of CD8^+^ and CD4^+^ T cells were observed in the spleen and peripheral blood mononuclear cells of treated mice. Collectively, these studies underscore the potential of AuNPs in conjunction with the cancer immune checkpoint blockade as a promising platform for combined photothermal therapy and immunotherapy.

#### 5.1.4. PTT-Based Combinatorial Treatments

In addition to adjuvants, antibodies, and immune checkpoint blockades, AuNPs can be combined with various drugs and site-specific receptors to facilitate targeted PTT immunotherapy. Notably, studies by Nam et al. have demonstrated that combining PTT with chemotherapy provides a robust platform for enhanced cancer immune therapy [119]. For instance, polydopamine-coated spiky AuNPs loaded with doxorubicin exhibited significant anti-tumor efficacy, achieving an impressive 85% survival rate in a CT26 colon carcinoma model. PTT studies revealed an augmented AH1-specific CD8^+^ T-cell response, attributed to the ability of doxorubicin to induce DC maturation and the synergistic effect of PTT with AuNPs. Moreover, hyaluronic acid (HA) conjugated to AuNPs enables the targeted delivery of both nanoparticles and drugs to cancer sites, enhancing PTT efficacy [120,121]. The interaction between HA and CD44 receptors facilitates the increased uptake of AuNPs, thereby improving PTT efficiency. Additionally, AuNP-based nanovaccines incorporating HA have shown promising results in stimulating DCs to produce CD8^+^ T cells for cancer immunotherapy [74]. Further advancements include the development of Au nanostar@CaCO_3_/Ce6 nanoparticles, which, when loaded into human NK cells, exhibit an enhanced photothermal response and reactive oxygen species formation [122]. The nanocomplexes demonstrate target specificity, stability, and improved biocompatibility, leading to increased cytokine levels in tumor cells upon NIR irradiation. This combined approach has yielded promising results in both in vitro and in vivo studies, highlighting the potential of AuNPs-PTT and AuNPs-PTT-chemo combinations as therapeutic strategies in cancer immunotherapy.

The application of AuNPs using their photothermal effect in cancer immunotherapy is described in Table 2.

### 5.2. Application of AuNPs for PDT-Based immunotherapy

PDT is a minimally invasive cancer ablation technique in which a photosensitizer (organic or NPs), with the aid of light, produces cytotoxic reactive oxygen species to the tumor cells. Due to the intense light absorption and stability of NPs, especially AuNPs, PDT studies based on AuNPs find application in many tumor ablation studies such as breast, skin, colorectal, etc. [11,123]. PDT can also induce an immune response in cells by releasing tumor-associated antigens and cytokines, and enhance the proliferation of T cells to initiate the immunogenic cell death [44,124,125].

#### 5.2.1. PDT-Based Immunogenic Cell Death

PDT induces oxidative stress in tumor cells. This oxidative stress induces the secretion of tumor-associated antigens and DAMPs to stimulate the immune response. Studies showed that a hypoxia condition in tumor cells always decreases the efficiency of PDT treatment, and AuNPs can enhance the generation of ROS via their high optical absorption cross-section, which depends on their size and shape. AuNPs can be used as a photosensitizer or a carrier for the photosensitizer in PDT studies.

**Figure 9.**
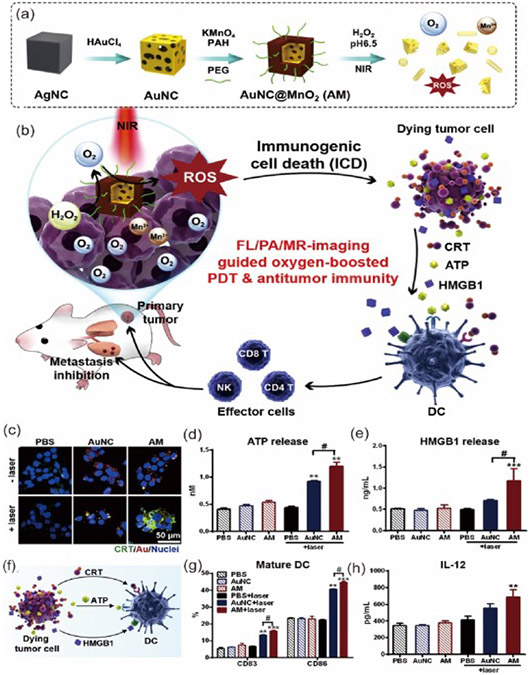
Working principle of the AuNCs@MnO_2_ (AM) nanomaterial for ICD, (**a**) A synthetic route for the preparation and generation of O_2_ from AM. (**b**) Therapeutic application of AM for the generation of cytokines and imaging studies. (**c–e**) Detection of various ICD signal molecules after AM + Laser treatment and PB is used as a control for the studies. (**c**) Fluorescence microscopic images for CRT expression in 4T1 cells. (**d,e**) Released ATP and HMGB1 in the supernatant after the AM + Laser treatment. (**f**) Schematic representation for the DC activation by ICD. (**g**) The expression for CD83 and CD86 after DC maturation. (**h**) The generated IL-12 in the culture supernatant. The asterisks indicate differences between PBS and other treatments are statistically significant. ** *p* < 0.01, *** *p* < 0.001. # Differences between the two groups are statistically significant; # *p* < 0.05. (n = 5). Reproduced with permission from [126], Copyright 2018, Elsevier.

Liang et al. showed that a hollow Au nanocage (AuNCs) with a MnO_2_ layer forms a core-shell nanoparticle that can generate molecular oxygen in the acidic tumor environment (Figure 9a) [126]. Since the tumor cell contains a large amount of H_2_O_2_, it reacts with MnO_2_ to generate O_2_ (MnO_2_ + H_2_O_2_ + 2H^+^ →Mn^2+^ + 2H_2_O+O_2_↑). An added advantage of this nanoparticle system is that, due to the presence of Mn^2+^ ions and released oxygen, the system can be used for fluorescence/photoacoustic/ MRI imaging studies to diagnose cancer (Figure 9b). This reaction enabled the generation of a large amount of ROS in the tumor environment to enhance the PDT to improve the ICD by generating calreticulin (CRT), adenosine triphosphate, and high mobility group protein B1 (HMGB1) (Figure 9c-e). The nanomaterial induced DC maturation and activated the T-cell response in metastatic triplenegative breast cancer by generating CD8^+^ T cells, CD4^+^ T cells, and NK cells. Together with NPs and laser treatment displayed the highest proportion of CD8^+^CD69^+^ T (11.0%), CD4^+^CD69^+^ T (9.9%), or NK1.1+CD69^+^ (7.5%) in tumors, indicating its strong ability to increase activated effector cells (Figure 9f-h). Thus, the presented nanosystem induced immunogenesis through PDT-based tumor treatment via ICD. In a study conducted by Chang et al., they found that modifying copper nanosheets with AuNPs initiated the reaction of H_2_O_2_ to generate O_2_ [127]. This hybrid structure not only enhanced the efficacy of PDT by alleviating tumor hypoxia compared to copper nanosheets alone but also boosted cytokine secretion and dendritic cell maturation. These effects elicited immune responses and fostered a memory effect of CTLs, ultimately suppressing tumor metastasis.

#### 5.2.2. PDT and Immunoadjuvant Cancer Therapy

Combining the phototoxic ROS generation and adaptive immune responsiveness of the tumor cells due to PDT is a good strategy for designing various cancer immune therapeutic agents. Since the conjugation of immune checkpoint blockades and adjuvants to AuNPs can enhance the immunotherapeutic efficiency along with PDT, the combination of immunoadjuvants can increase the infiltration of DCs and activate the CD8^+^ T cells.

Marrache et al. prepared an Au nanocomplex system for PDT and cancer immunotherapy in 4T1 cells [128]. Zinc phthalocyanine, which can absorb light in the 600–800 nm range, has been used as a photosensitizer and conjugated with poly (D, L-lactic-co-glycolic acid)-b-poly(ethylene glycol) as a drug carrier. The polymer layer is coated with AuNPs, and the adjuvant CpG-ODN is conjugated with the AuNPs for targeted tumor therapy. The in vitro phototoxicity studies in 4T1 cells showed that irradiation using a 660 nm laser induced significant cell death at IC50 of 2.8 nM of the nanocomplex. Studies showed that the toll-like receptor 9 recognizes the CpG-ODN in the nanocomplex and induces the significant enhancement in the production of IL-6, IL-12, IFN-α/β, and TNF-α. Thus, AuNPs induced stress in the endoplasmic reticulum (ER), and, along with PDT, the controlled release of CpG-ODN occurred, followed by DC maturation and T-cell activation. This study opens a new path for photoimmunotherapy using AuNPs for metastatic cancers. In one of the recent studies, Lin et al. showed that a combination of Au and upconversion nanoparticles decorated with ethylenebis(nitrilodimethylene)tetraphosphonic acid can be used for the real-time dynamic imaging of mitochondria. Moreover, an assembly of these NPs with zinc-phthalocyanine-induced and anti-CTLA-induced immune response and enhances the photodynamic therapy to kill cancer cells [129].

#### 5.2.3. Combined PDT and PTT Cancer Immunotherapy

While PTT and PDT have long been recognized for their phototherapy applications in cancer treatment, they are not without limitations, including issues related to target specificity, deep tissue penetration, and tumor recurrence. Combining PTT and PDT with AuNPs in cancer immunotherapy presents an intriguing avenue of research. For instance, Liu et al. demonstrated in a study the potential of a biocompatible gold nanocluster derived from captopril, Au25(Capt)18, which exhibited a remarkable photothermal stability and an enhanced generation of singlet oxygen [130]. Their research indicated that, in cutaneous squamous cell carcinoma, PTT contributed to 28.86% of cytotoxicity, while PDT contributed to 71.14%. Moreover, together with the nanocluster and NIR laser, it revealed a significant increase in the production of CD4^+^ T and CD8^+^ T cells at 44 ^◦^C with an irradiation time of five minutes. In another study, Jin et al. showcased the efficacy of a corn-like Au/Ag nanorod conjugated with CTLA4 under NIR laser irradiation, which induced simultaneous PDT and PTT therapy along with eliciting a cancer immune response in 4T1 tumor cell lines [131]. Importantly, this approach led to the generation of long-term immune memory in mice, evidenced by the presence of CD3^+^, CD8^+^, CD62L−CD44^+^ T cells.

The induction of endoplasmic reticulum (ER) stress serves as a crucial step in initiating ICD through the utilization of NPs. Many photosensitizers (PSs) employed in this process specifically target the ER, triggering the production of reactive oxygen species and subsequent ER stress. This cascade ultimately leads to cellular destruction and the activation of the ICD pathway. In a recent study by Li et al., a novel nanomaterial combining PDT and PTT was developed [132]. This nanomaterial consisted of hollow gold nanospheres functionalized with an ER-targeting pardaxin peptide linked to indocyanine green, along with oxygen-delivering hemoglobin liposomes to mitigate tumor hypoxia. Upon light irradiation, ER stress was induced, accompanied by the upregulation of calreticulin, a recognized biomarker for ICD. Consequently, DC maturation ensued, initiating a cascade of immune responses within the CT-26 tumor model, leading to the proliferation of CD8^+^ T cells and secretion of cytokines essential for an effective anti-cancer immune response. Similarly, in the B16 tumor model, enhanced DC maturation was observed, resulting in the increased secretion of CD11c^+^/CD80^+^/CD86^+^ T cells. Various Au nanostructures used for PDT-based immunotherapies are listed in Table 3.

## 6. Radiation Immunotherapy

Radiation therapy (RT) is one of the minimally invasive techniques that has been applied to many cancer patients in the acute stage [133,134]. This technique uses suitable radiation sources, primarily radioactive elements, which generate highly intense X-rays to damage the tumor cells. One of the issues with RT is that it can damage not only the tumor cells, but also the surrounding healthy tissues. Additionally, the dosage of radioactive elements is high, which can impart certain side effects after exposure [135,136].

Metal radioactive sensitizers possess an excellent absorption coefficient, especially AuNPs with higher atomic numbers [137,138]. Auger electrons produced after the radio sensitization of AuNPs can generate ROS effectively [44]. Due to the excellent surface modification properties of AuNPs, one can prepare target-specific nanomaterials for RT either passively or actively [139,140]. Since AuNPs exhibit an excellent EPR effect, they enable the low dosage of the radioactive element compared to the existing materials. For example, in one study, Chattopadhyay et al. showed that the conjugation of trastuzumab to PEG-AuNPs had a targeted specificity toward SK-BR-3 human breast cancer cells and induced an enhanced DNA double-strand breakage (5.1 times) compared to RT and RTcombined PEG-AuNPs alone using 300 kVp X-rays [141].

RT-induced ICD accelerates the secretion of many immunogenic antigens and cytokines, and eventually leads to T-cell activation. Additionally, the immunogenic response secretes ATP, HMGB1, and CALR to activate the signals for DC maturation and T cells. Janic et al. studied the cellular uptake of AuNPs with two different sizes, 4 and 14 nm, and checked their efficiency for RT in an MDA-MB-231 xenograft mouse mode [139]. Studies showed that both 4 and 14 nm possessed excellent cellular uptake, with 4 nm AuNPs exclusively located in the cytoplasm and 14 nm particles in the nucleus determined by transmission electron microscopy. RT+AuNPs showed enhanced tumor growth delay, compared to the control, RT, and AuNPs alone. The 14 nm-sized AuNPs showed an increased survival rate with enhanced ICD and macrophage infiltration compared to the 4 nm-sized AuNPs with a dosage of 100 μg per tumor. The ER stress effect of AuNPs initiated the immune response and the RT-induced release of DAMPs and CALR. The difference in ICD for AuNPs with different sizes is unclear yet, but this multimode RT immunotherapy can be used for future cancer immunotherapy applications.

Using suitable and targeted dosages of radiosensitizers can overcome the adverse side effects of RT on the surrounding tissues. One such strategy in cancer immunotherapy is using immune checkpoint blockades along with radiosensitizers. Cao et al. showed that cytosine-phosphate-guanine oligodeoxynucleotide (CpG ODN)-adjuvant-conjugated AuNPs can regulate the tumor-associated macrophages and enhance the RT and immune checkpoint blockades, thereby enhancing the secretion of cytokines [142]. Studies in the bilateral glioma (GL261) tumor model showed that synergy between CpG-AuNPs and α-PD-1 induces macrophage infiltration, the blockade of PD-1, and the activation of the adaptive immune response after RT with a low-dose X-ray application. Thus, the presented nanosystem can be used as a potent strategy for RT-based immune checkpoint blockades and cancer immunotherapy.

The AuNPs with a photothermal and high X-ray absorption ability can be effectively utilized for cancer immunotherapy. Ma et al. showed that Au nanospike-like structures can be used for photothermal tumor ablation studies with a photothermal conversion efficiency of 50% in human oral epidermoid carcinoma (KB) cells [143]. Both in vivo and in vitro studies showed that combining Au-nanospikes with NIR laser and X-ray treatment could induce DNA damage, ROS generation, apoptosis, and necrosis in the tumor cells. The nanocomplex showed 1.4-fold DNA damage, with a 2-fold increase in ROS, and a 17.92% increment in apoptosis in the combined therapy and opened a new design strategy for cancer immunotherapy.

**Figure 10.**
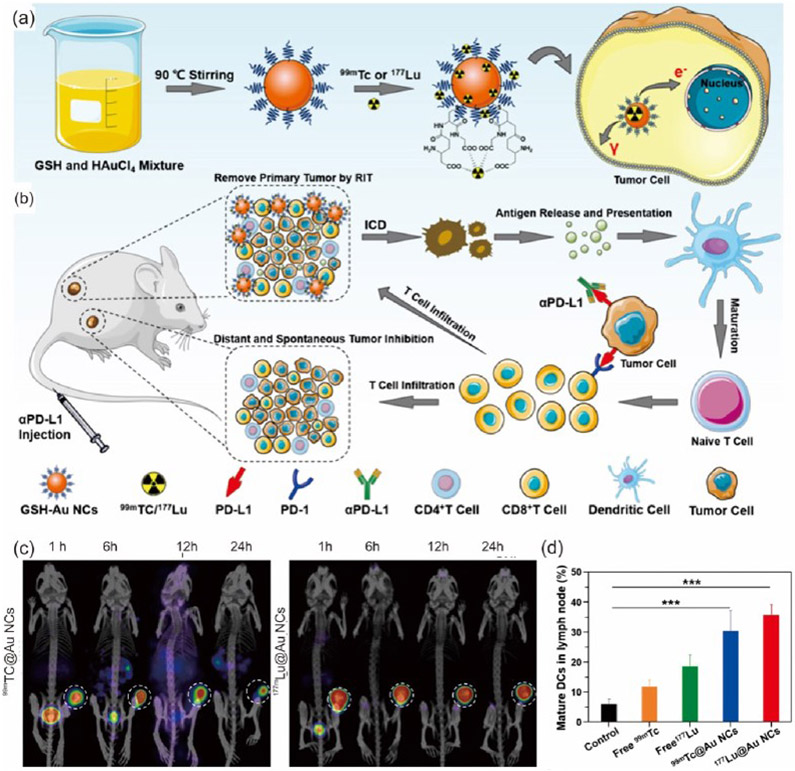
(**a**) A schematic representation for the glutathione-based AuNC formation and complexation of 177Lu or 99Tc. (**b**) Cartoon diagram showing the cancer immunotherapy pathway and the immune checkpoint blockade pathways. (**c**) The internalization and SPECT/CT images for 99Tc and 177Lu@GSH-AuNCs in 4T1 tumor-bearing mice after regular intervals of time. (**d**) represents the DC maturation in CT26 cells using the various radionuclides and the 99Tc and 177Lu@GSH-AuNCs, where *p* values were calculated by multiple *t*-tests (*** *p* < 0.001). Reproduced with permission from [144], Copyright 2021, Elsevier.

A radioactive nucleus with a long half-life can be combined with AuNPs for cancer immunotherapy. In such cases, Au can act as a targeted drug carrier with efficient photothermal properties, and the radioactive nuclei can enhance the RT. In one recent study, Pei et al. prepared glutathione-coated Au nanoclusters (GSH-AuNCs, 2.5 nm) conjugated with ^177^Lu or ^99^Tc by the carboxyl groups on the surface of GSH [144] (Figure 10a). The conjugation of Lu or Tc with AuNCs enhanced their radio-sensitization property, compared to the free lanthanides (Figure 10b). SPECT/CT images showed that, due to the excellent EPR effect of AuNCs, the lanthanides accumulated in the tumor sites specifically (4T1 cells), with enhanced blood circulation. In vivo radioisotope therapy in colorectal cancer CT26 tumors showed that both ^177^Lu and ^99^Tc@GSH-AuNCs showed approximately 35.65% and 30.4% DC maturation, respectively (Figure 10c). Meanwhile, the free ^177^Lu or ^99^Tc did not induce any DC maturation. The synthesized nanocomplex showed excellent ICD along with αPD-L1 blockade efficiency (41.93%), and enhanced CD3^+^, CD8^+^, CD62L−CD44^+^ T-cell activation. ELISA kit studies showed increased TNF-α and IFN-γ secretions in the serum. Thus, the present radiolabeled AuNCs can be used as a potent therapeutic agent for radio-immunotherapy.

The present studies showed that AuNPs alone or in combination with various radionucleotides can enhance radiation therapeutic efficiency. AuNPs can be used as carriers to deliver radionucleotides into tumor sites with specificity, which is otherwise impossible. Soon, we can expect more promising research work in RT-induced cancer immunotherapies.

## 7. Sonodynamic Immunotherapy

Even though many advanced therapies such as chemo, RT, PTT, and PDT, in combination with cancer immunotherapies have been developed, the side effects of chemo/RT and the low penetration effect of PTT/PDT are still drawbacks of these therapies. Sonodynamic therapy (SDT), using sonosensitizers with ultrasound waves (US), can achieve deep tissue penetration even up to a 10 cm depth [145,146]. In SDT, ultrasound waves produce ROS with monosensitization and minimal adverse effects on the normal tissues. Thus, the combination of SDT with cancer immunotherapies can bring promising results and strategies in cancer immunotherapy compared to the existing therapeutic techniques. In recent years, researchers have been trying to develop various sonosensitizers from organic and inorganic materials, but organic materials possess stability issues and low pharmacokinetics compared to inorganic materials [147]. Due to the advancement in nanotechnology, many inorganic NPs (Ag, Au, Ti, Cu, etc.) are currently used as a sonosensitizer for the generation of ROS [148-150]. The unique surface functionalization property and biocompatibility of AuNPs compared to the other noble metals enable AuNPs to be widely used in SDT. Even though the exact mechanism of SDT is not known, it is assumed that the cumulative ROS generation and thermal and mechanical effects are responsible for the therapeutic outcome. Studies showed that AuNPs alone or in combination with other metallic NPs can improve the ROS and SDT efficacy. Goncalves et al. showed that PEG-coated AuNPs conjugated with δ-aminolevulinic acid (18 nm size) can be used as a sonosensitizer for SDT [151]. The SDT induced an 87.5% reduction in macrophage viability by pulse ultrasound irradiation (1 W/cm^2^ with 1.0 MHz) within 2 min. In another study, Deepagan et al. showed that the ROS generation and SDT efficiency in TiO_2_ NPs can be improved by adding Au into the nanocomposite [152]. Studies on SCC7 tumor-bearing C3H/HeN mice showed that the nanocomposite with ultrasound radiation showed a 3.11-fold higher tumor regression than HTiO_2_ NPs. The ROS generation can activate the generation of cytokines, immune anti-gens, maturation of DCs, and T cells for cancer immunotherapy. A variety of hybrid Au nanostructures were reported recently for the synergic PTT and SDT studies where a combination of various heterometallic structures improved the SDT applications for tumors [153-156]. A hybrid nanoparticle system (Au-BPNs) was synthesized by Ouyang et al. by the in situ reduction of Au on black phosphorous quantum dots. The layered structure of BP facilitates the interaction of O_2_ to form ^1^O_2_, which enhances tumor ablation with fewer side effects by ultrasound irradiation [157]. The generation of ROS using US was found to be 4.7 times higher than 660 nm NIR light. Histological and flow cytometry studies showed that the ratio of apoptotic cells is higher by SDT treatment than by PDT treatment. Cao et al. fabricated Au nanocrystals on the edges of TiO_2_ nanosheets for targeting mitochondria [158]. The Au-TiO_2_ material underwent additional modification with mitochondria-targeted triphenylphosphine (TPP) and AS1411 aptamer, resulting in Au-TiO_2_-A-TPP. This modification was aimed at achieving enhanced SDT and CT imaging targeted at organelles. Both in vivo and in vitro studies showed a sufficient generation of ROS within the mitochondria.

Studies have shown that SDT and a combination of other therapies can induce ICD. Other than the CpG adjuvant PD-1/PD-L-1 blockade toll-like receptor activation, inhibiting the expression of indoleamine 2,3-dioxygenase (IDO) is another strategy in cancer immunotherapy. With the help of nanotechnology, a combination of SDT and IDO has recently been adopted for cancer immunotherapy. In one of the recent studies by Zhang et al., a biomimetic system is used for metastatic tumor treatment using a combination of SDT, CO gas generation, and IDO-based immunotherapy [159]. The sonosensitizer is loaded with a CO-releasing molecule, CORM-401, and coated with a macrophage cell membrane for effective tumor-targeting capability. US irradiation (1 MHz, 1 W/cm^2^) induced ^1^O_2_ generation along with CO release from the nanohybrid system. In vitro and in vivo studies in 4T1 and J774A-1 cells showed cell apoptosis and mitochondrial dysfunction. In vivo studies in 4T1 tumor cells using a synergy between SDT/CO gas therapy largely showed tumor suppression and a combination of IDO inhibitor NLG919-induced ICD. Under US irradiation, the calreticulin level in 4T1 cells increased along with the DC maturation (CD11c^+^ CD80^+^ CD86^+^) and T-cell activation. Thus, the combination therapy largely suppressed the tumor recurrence, and tumor metastasis was prevented.

Therefore, AuNP-based SDT is a promising strategy for cancer immunotherapy. Although there are many examples of AuNPs being used to generate ROS and act as a sonosensitizer, the studies are preliminary. For clinical trial applications, many parameters must be optimized in detail. The detailed mechanism of SDT is unclear in the biological systems, and the cancer TME is also a big challenge in clinical trial applications. Table 4 categorizes various AuNPs recently used for the SDT tumor therapy.

## 8. Conclusions and Future Directions

The optical and chemical properties of AuNPs can be adapted well for cancer immunotherapy studies. The excellent tenability of the size and shape of AuNPs, which, in turn, alters their photosensitization and drug-loading efficiency, can benefit the cancer immunotherapy field as drug carriers or as thermal agents in immunotherapies. Cancer immunotherapy has revolutionized treatment methods for tumors in recent years. The treatment is based on the activation of the human immune-responsive nature towards cancer cells. Compared to other existing methods like surgery, PTT, and PDT, chemotherapy, immunotherapy offers minimal side effects since the treatment acts as a checkpoint between the innate and acquired immune response of the cells. However, one of the major problems in cancer immunotherapy is the unwanted activation of T cells, and, sometimes, the drug molecules can create side effects during long-term use. Target-specific therapy is still challenging, and, to overcome this, nanotechnology-based drug carriers can be used. AuNPs are especially known for their excellent biocompatibility, good capability as a drug carrier, stimuli-responsive nature, and, most importantly, the surface functionalization property for target-specific actions. Recently, AuNPs have been used as carriers for various cancer immunotherapeutic agents, and to activate T cells even without any immunotherapeutic agents. AuNPs allow the target-specific release of immunotherapeutic agents, and combining their unique features with other treatment methods like PDT, PTT, SDT, and RT can create a plethora of platforms for cancer treatment agents.

Even though nanotechnology offers great improvements in cancer immunotherapybased treatments with its lowered drug dosage and minimal cytotoxicity, these treatments are still expensive when they come from a clinical point of view. Additional efforts should be devoted to enabling Au nanomedicines to be a cost-effective alternative for many existing systems. The precise target specificity, long-term stability, usage doses, and side effects have also been considered for wider clinical applications. Even though many of the existing studies are limited to in vitro, for in vivo studies, one must take care of the aspects of tumor heterogeneity since the composition and environment for tumor cells vary from tumor to tumor and person to person.

A combination of various nanoformulations like liposomes, and poly(lactic-co-glycolic acid) (PLGA), along with AuNPs can improve and reduce the cytotoxic effects of AuNPs, which are size-dependent. There are several reports in which PLGA can be used for the maturation of DCs and can show anti-PD-L1 properties [160]. Similarly, liposomes can be used to transfer genes for cancer immunotherapy [161]. Therefore, the combination of these nanoformulations with AuNPs can create new composite materials for cancer immunotherapy. Moreover, the photosensitization and photothermal properties can also stimulate the immune response in cancer cells by the secretion of heat-shocking proteins to navigate the immune response in tumor T cells.

Moreover, the translation of AuNP-based immunotherapies from preclinical models to clinical settings necessitates rigorous validation and optimization. Robust clinical trials are imperative in order to assess the safety, efficacy, and long-term outcomes in diverse patient populations. Furthermore, the development of scalable manufacturing processes and quality control standards is essential for facilitating clinical translation and ensuring consistent therapeutic efficacy. In short, while the application of AuNPs for cancer immunotherapy represents a burgeoning frontier with immense therapeutic potential, addressing key challenges and unanswered questions is imperative in order to realize their clinical impact fully. Through an interdisciplinary collaboration and systematic investigation, the refinement of AuNP-based immunotherapies holds promise in revolutionizing cancer treatment paradigms and improving patient outcomes.

## Figures and Tables

**Table 1.** Application of gold nanostructures and their therapeutical clinical use.

Name of the Drug	Nanoparticle Type Used	Clinical Trial	Applications
Gold nanoshells	60–70 nm silica core and 15–40 nm gold shell	NCT01270139, Completed (2007–2016)	For the photothermal ablation of atherosclerotic plaques
CNM-Au_8_	13 nm AuNP dispersed in drinkable bicarbonate solution	NCT02755870, Phase I, completed (2015–2016) NCT03536559, Phase II, Active (2018–); NCT03815916, Phase II, completed (2019–2021); NCT03993171, Phase II, recruiting (2019–); NCT04081714, Available (2019–); NCT04098406, Phase II, Completed (2019–2021); NCT04414345, Phase II/III, Active (2020–); NCT04626921, Phase II/III, Active (2020–); NCT05299658, Phase II, Active (2021–)	For the treatment and curing of various neurodegenerative disorders
Auroshell	PEG-functionalized silica core (120 nm) with gold shell of 10–15 nm	NCT00848042 NCT01679470	For the photothermal therapy of various tumors, prostate, neck, and head
NU-0129	13 nm AuNP coated with SiRNA and-SH-PEG	NCT03020017, Phase 0, Completed (2017–2020)	For glioblastoma
CYT-6091	TNF-functionalized 27 nm AuNP with PEG	NCT00356980, Phase 1, Completed (2006–2009) NCT00436410, Phase 0, Completed (2006–2009)	Tumor therapy by regulating immune response
naNO-COVID	Cocktails of peptides from the coronavirus, tethered to the surface of AuNP for T-cell priming	NCT05113862, Phase I, Active (2022–)	Vaccine against COVID
naNO-DENGUE	AuNP surface-bound cocktails of peptides from the dengue virus for priming T cells.	NCT04935801, Phase I, Active (2021–)	Vaccine against dengue
C19-A3 AuNP	The human proinsulin peptide (C19-A3) linked to ultrasmall AuNP (<5 nm)	NCT02837094, Phase I, Active (2016–)	Managing autoimmune disorder type 1 diabetes.

**Table 2.** List of AuNP structures studied for photothermal immunotherapy.

Photothermal Nanoparticles	Immunoadjuvants or Checkpoint Blockade	Effector Cells	Cytokines	Tumors	References
BSA-AuNRs	R837	DCs, CD8^+^ T-cells	TNF-α, IL-6, IL-12	Murine melanoma cell B16-F10	[109]
AuNSs	CpG	DCs, CD8^+^ T-cells, CD4+ T-cells	IL-2, IL-6, IFN-γ	Murine gastric cancer cell MFC	[110]
AuNR-PEI	CpG	DCs, CD8^+^ T-cells, CD4^+^ T-cells	-	Murine breast cancer cell 4T1	[111]
AuNR-DNA hydrogels	CpG	-	TNF-α, IL-6, IL-12p40, IFN-γ	Murine T lymphoma cell EG7-OVA	[112]
AuNSTs	Anti-PD-L1	CD8^+^ T-cells, CD4^+^ T-cells, B cells	-	Murine bladder cancer cells MB49	[114]
AuNSTs	Anti-PD-L1	CD45), (CD3), CD4, CD8, and T regulatory cells (CD4/CD25/FOXP3)	-	Brain tumor	[115]
Au@Pt NPs	Anti-PD-L1	CD8^+^ T-cells, CD4^+^ T-cells	TNF-α, IL-6, IL-12p70, IFN-γ	Murine breast cancer cell 4T1	[116]
AuNCs	Anti-PDL1	CD11c, CD80, CD11c CD86	-	Hepatocellular carcinoma	[117]
HAuNS	Anti-PDL1	DCs, CD8^+^ T-cells	TNF-α, IL-2, IL-12p70, IFN-γ	Murine breast cancer cell 4T1, murine colon cancer cell CT26	[118]

**Table 3.** List of AuNP structures for photodynamic immunotherapy.

Nanoparticles	Photosensitizers (PSs)	Effector Cells	Cytokines	Tumors	References
AuNCs	MnO_2_	DCs, CD8^+^ T-cells, CD4^+^ T-cells, NK cells	IL-12	Metastatic triple breast cancer	[126]
AuNPs	Tetraphenylethylene	DCs, CD86, CD80	IL-2, IL-6, IL-12, TNF-α, IL-10	B16F10 tumor-bearing mice	[127]
AuNP/CpG-ODN	Zinc phthalocyanine	DCs, CD8^+^ T-cells, CD4^+^ T-cells	IL-6, IL-12, IFN	4T1cells	[128]
Au nanocluster	-	CD8^+^ T-cells, CD4^+^ T-cells		Cutaneous squamous cell carcinoma	[130]
Au/Ag nanorod + CTLA4	-	CD3^+^CD8^+^CD62L−CD44^+^ T cells	TNF-α and IFN-γ	4T1 tumor cell lines	[131]
Au nanosphere	Indocyanine green	CD11c^+^/CD80^+^CD86^+^ T cells	TNF-α and IFN-γ	B16 tumor model	[132]

**Table 4.** Represents a few examples of AuNPs used for SDT recently.

Sonosensitizers	Mechanism	Mode of Action	In Vitro/In Vivo US Parameter	In Vivo/In Vitro	Ref
Au-MnO	ROS	CDT+SDT	1 MHz, 2 W/cm^2^, 10 min	orthotopic liver tumor	[153]
Au NPL@TiO_2_	ROS	PTT+SDT	3 MHz, 0.5 W/cm^2^, 20 min	Hela cell line	[154]
Au NPs	acoustic cavitation	US therapy	1.1 MHz, 2 W/cm^2^, 3 min	CT26 cell line	[155]
Au-PPIX NPs	ROS and cavitation	SDT	1.1 MHz, 2 W/cm^2^, 3 min	CT26 cell line	[156]
Au@BP NPs	ROS	SDT	1 MHz, 1 W/cm^2^, 3 min	4-T1 cell line	[157]
Au-TiO_2_-A-TPP	ROS	SDT+CT	1.0 MHz, 1.5 W/cm^2^, 5 min	MCF-7	[158]

Abbreviations: CDT: chemodynamic therapy, BP: Black phosphorous, NPL: nanoplate, PPIX: Protoporphyrin IX, A: AS1411 ap-tamer, TPP: mitochondria-targeting triphenylphosphine, CT: computed tomography.
